# Derivation and Comprehensive Analysis of Aging Patterns in Patients with Bladder Cancer

**DOI:** 10.1155/2021/3385058

**Published:** 2021-10-21

**Authors:** Bin Wang, Fachun Tong, Chengxi Zhai, Long Wang, Yunzan Liu, Jian Wang

**Affiliations:** Department of Urology, People's Hospital of Yuxi City, Yuxi City, Yunnan Province, China

## Abstract

**Background:**

Aging is an essential risk factor for cancer. However, aging-related genes (ARGs) have not been comprehensively analyzed in bladder cancer (BC). Therefore, the study is aimed at derivating a risk stratification system for BC patients based on ARGs.

**Methods:**

Public databases were used to acquire ARGs sets, transcriptome files, and clinical data. The “limma” package was then used to screen for differential ARGs while also using univariate Cox regression analysis to explore for prognostic ARGs. The “ConsensusClusterPlus” package was used to perform aging patterns in BC patients based on the above prognostic ARGs. Subsequently, aging patterns were investigated in survival prediction, mutation landscape, immunotherapy, immunological checkpoints, and immune microenvironment. We likewise utilized gene enrichment analysis to explore the biological functions that were behind the findings. To construct a risk signature and nonogram for prognostic prediction, we used LASSO and Cox regression analysis based on differential genes in aging patterns. In addition, we plotted a nomogram and validate the accuracy of the risk signature in GEO and TCGA cohorts. We explored the possible biological mechanism using GSEA analysis and preliminarily identified a hub gene using PPI network. Finally, we validated the expression of hub gene in BC cell lines.

**Results:**

We screened 84 downregulated ARGs, 74 upregulated ARGs, and 32 prognostic ARGs in the human aging genome resource. The aging patterns based on prognostic genes had excellent survival prediction (*p* < 0.001) and discriminatory ability in 405 BC patients. In addition, we found no significant differences in aging patterns in mutation analysis, which were all characterized by *TP53*, *TTN*, and *KMT2D* mutations. It is worth noting that cluster B in the aging patterns has a better response to immunotherapy and a more active immune microenvironment (*p* < 0.05). In addition, gene enrichment analysis showed that aging patterns may be related to biological processes such as Staphylococcus aureus infection, phagosome, and cytokine-cytokine receptor interaction. Subsequently, we constructed a risk signature based on 16 differential genes from different aging patterns and had good survival prediction ability in both GEO and TCGA cohort. Specifically, survival analysis revealed a significantly shorter survival time in the high-risk group than in the low-risk group (TCGA and GEO, *p* < 0.001). In addition, AUC values in the ROC analysis predicted 1, 3, and 5 years in TCGA cohort that are 0.713, 0.714, and 0.738, respectively. AUC values predicted 1, 3, and 5 years in GEO cohort that are 0.606, 0.663, and 0.718, respectively. There is no doubt that risk score was an independent prognostic factor from results of multivariate Cox regression analysis in BC patients (*p* < 0.001). There were also significant differences in immune cell infiltration, immune checkpoint, and immune score between the two groups (*p* < 0.05), but it should not be ignored that the correlation with the HLA expression was weak. Finally, we identified and validated *CLIC3* as a hub gene that may be involved in the Wnt signaling pathway, etc.

**Conclusion:**

We provided robust evidences that aging patterns based on ARGs can guide targeted therapy and survival prediction in BC patients.

## 1. Introduction

Bladder cancer (BC) is one of the most prevalent genitourinary system cancers [1], and it can be divided into muscle-invasive and nonmuscle-invasive subtypes depending on infiltration. Notably, the incidence of BC is rising, and it ranks third among in male cancers [2]. Biomarker discovery and survival prediction are important topics in BC diagnosis across the world [3]. As a result, establishing an effective stratification method is critical for monitoring the survival status of BC patients as well as their treatment.

The human aging genome resource (HAGR) is a gene set database that uses a comprehensive analysis of the biology and genetics in the human aging process to screen hub genes related with aging, exposing aging-related genes (ARGs) as network hubs [4]. The gradual degradation of functions at the molecular and cellular levels is the most notable manifestation of aging [5]. The correlation between aging and cancer is becoming more obvious [6], and the primary markers of aging and cancer cell senescence have been investigated [7], with immunological senescence being one of the most prominent examples [8]. Meanwhile, bioinformatic-based methods were utilized to construct EMT-related [9] and immune-related [10] risk signature to predict overall survival in BC patients. However, aging-related risk signatures to predict survival status have never been established in BC patients.

Therefore, in this study, to assess the prognostic value of ARGs in BC, we constructed aging patterns and further established a risk signature to reveal the potential association of ARGs with immunotherapy and survival prediction.

## 2. Materials and Methods

### 2.1. Bioinformatic Datasets and Data Preprocessing

The BC-clinical data, BC-RNA sequencing profiles (*n* = 414), and normal bladder epithelium RNA sequencing profiles (*n* = 19) were obtained from The Cancer Genome Atlas (TCGA) database [11]. We excluded BC patients without RNA sequencing and survival time, and finally, only 405 patients were retained for subsequent analysis. In addition, we downloaded the GSE13507 dataset from the GEO database and included 165 patients with primary BC as an external validation cohort. Meanwhile, genes were identified based on annotation documents of the GENCODE database [12] and GPL6102. Finally, a total of 20634 common genes were annotated in the above two datasets. In addition, 307 PRGs were extracted based on previous studies [13].

### 2.2. Sample Classification for Aging Patterns

The “limma” package was used to screen for differential ARGs in normal and tumor tissues (∣logFC | >2 and *p* < 0.05). Prognostic ARGs (*p* < 0.05) were then screened using univariate Cox regression analysis. We performed two classifications using “ConsensusClusterPlus” package in *R* software. In the whole BC patients, the best *k* value was selected by identifying the inflection point of the sum of squared error (SSE) based on the prognostic ARG expression. After *k* = *i*, the rate of decline slowed down; so, *k* = *i* was chosen. In addition, we performed Kaplan-Meier survival analysis and PCA analysis on cluster A and B groups in aging patterns.

### 2.3. Construction of an Aging-Related Risk Signature and Nomogram

The “limma” package was used to screen for differential genes in cluster A and B groups (∣logFC | >2 and *p* < 0.05). Prognostic genes (*p* < 0.05) were then screened using univariate Cox regression analysis. These prognostic genes were further incorporated into multivariate Cox and LASSO regression analysis to identify genes involved in signature construction. We used the appropriate *λ* to build the model and to control the complexity of LASSO regression. The risk score was calculated as follows: risk score for OS =

(1) $$\begin{array}{c} \overset{n}{\underset{i=1}{\sum }}{\text{Coef}}_{i}\times x_{i}. \end{array}$$

Based on the coefficients of the above formula, we use “regplot” package to build a normograms. In addition, we performed Kaplan-Meier survival analysis, ROC analysis, and calibration curve for validating.

### 2.4. Functional Enrichment Analysis

Enrichment analysis was performed in differential genes in cluster A and cluster B groups using “ggplot2,” and “clusterProfiler” packages in *R* software. Gene Ontology (GO) analysis and the enrichment analysis of Kyoto Encyclopedia of Genes and Genomes (KEGG) were extracted from the result of “clusterProfiler” package. In addition, GSEA enrichment analysis was also conducted in different risk groups distinguished by risk signature.

### 2.5. Comprehensive Immune Analysis

In the exploration of differences in immune cell infiltration, we simultaneously used the CIBERSORT algorithm to estimate the abundances of immune cells in different risk groups distinguished by risk signature. Moreover, we used the estimation algorithm to calculate purity of tumor. More importantly, we also explored the expression levels of immune checkpoint and HLA-related genes in different risk groups.

### 2.6. Construction of PPI Network

To investigate interacting genes, genes involved in risk signature were imported into a STRING database (confidence = 0.900), which was used to predict the PPI network. We then selected the gene with the highest number of sides as the hub gene.

### 2.7. Assays

Two bladder cancer cell lines (5637 and UM-UC-3) and a normal bladder epithelium cell line (SV-HUC-1) were purchased from ATCC. The RNA expression was assessed by quantitative real-time PCR using the TB Green Premix Ex Taq II kit (TakaRa, Japan). Si-RNA targeting CLIC3 was purchased from Genepharm (Nanjing, China). Relevant antibodies were purchased from Santa Cruze (USA) and diluted at 1 : 1000. Detailed experimental details were carried out according to the methods in previous reference [14]. The sequences of the primers used for qRT-PCR are as follows: CLIC3 (forward: 5′-CAGATCGAGGACTTTCTGGAG-3′, reverse: 5′-GGAGAACTTGTGGAAAACGTC-3′) and GAPDH (forward: 5′-CAGGAGGCATTGCTGATGAT-3′, reverse: 5′-GAAGGCTGGGGCTCATTT-3′) [15].

### 2.8. Statistical Analysis

All statistical analyses were performed using the *R* software (v.4.0.1). Detailed statistical methods about transcriptome data are covered in Bioinformatics Method. *p* < 0.05 was considered statistically significant.

## 3. Results

### 3.1. Aging-Related Patterns Are Mediated by 32 Aging-Related Genes in BC Patients

**Figure 1:**
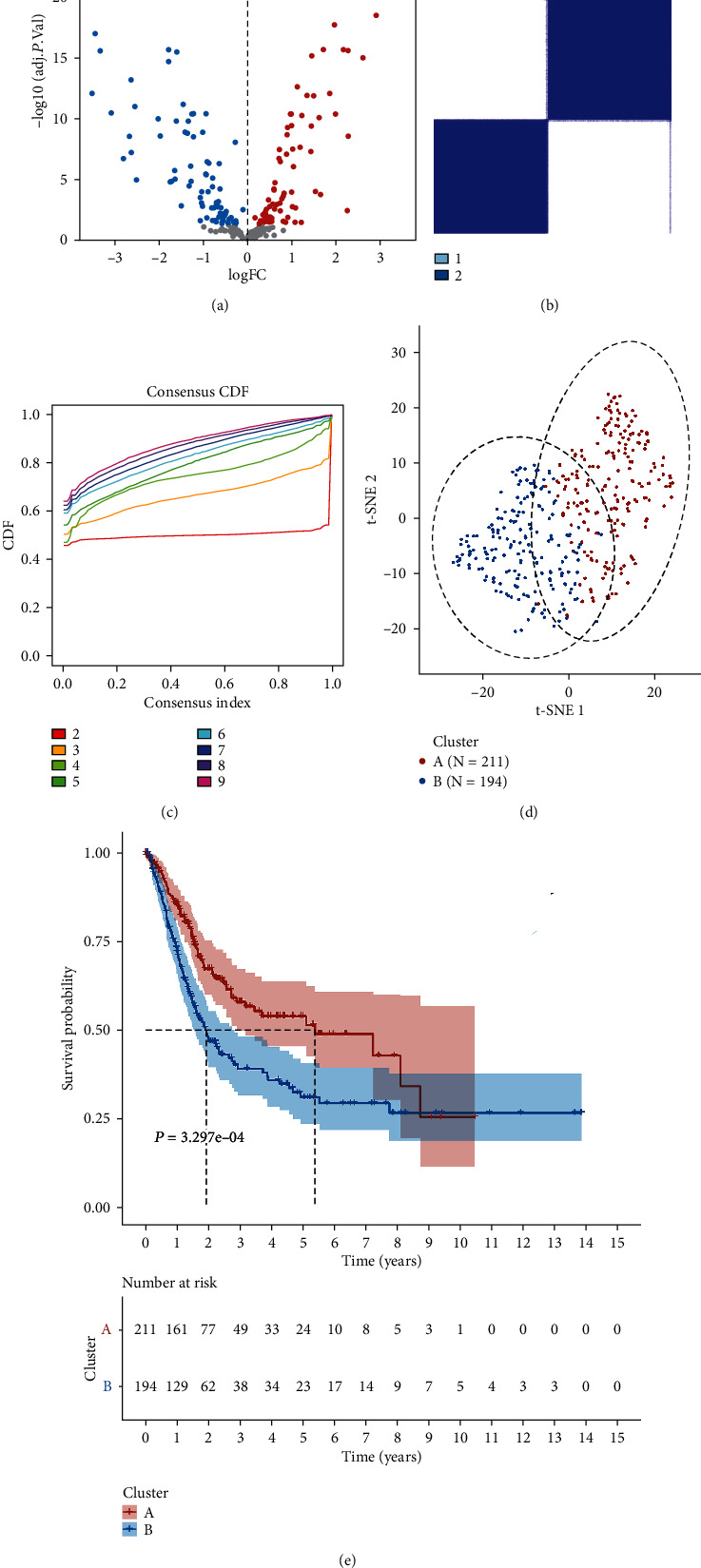
Consensus clustering for aging-related patterns. (a) The volcano plot for the ARGs in BC patients. Black dot, blue dot, and red dot represent no statistical significance genes, low expression genes, and high expression genes, respectively. (b, c) Consensus clustering identified two subgroups according to the expression of prognostic ARGs. (d) PCA analysis, including cluster A and cluster B. (e) Kaplan-Meier survival analysis in different clusters.

**Figure 2:**
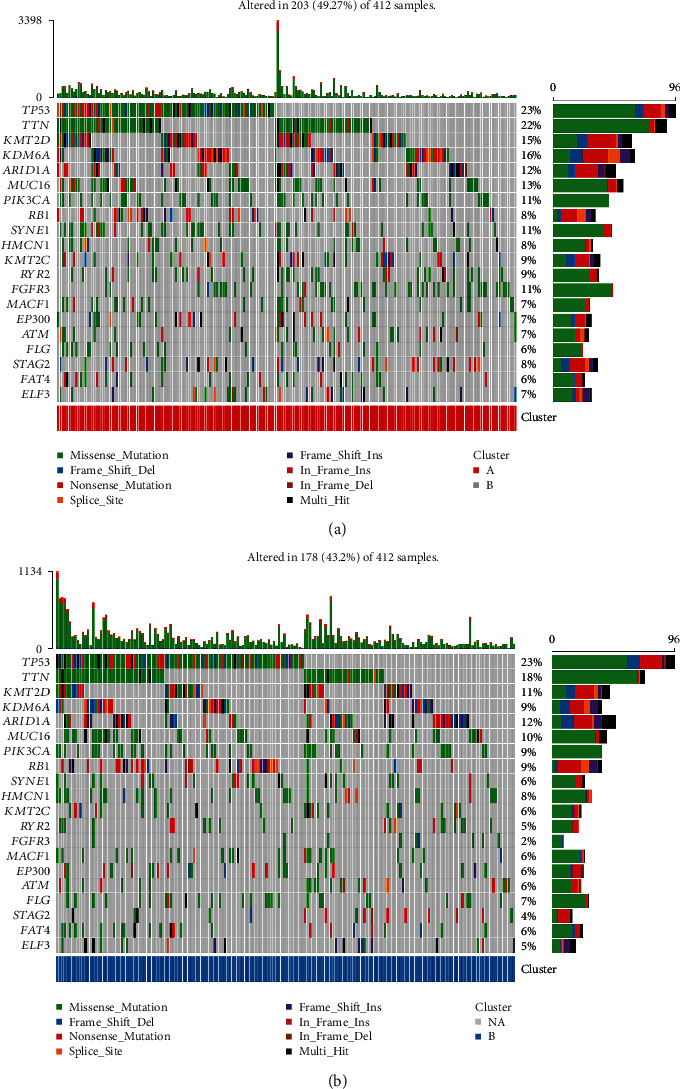
Mutation analysis in different aging-related patterns. (a) Mutation frequency of different genes in cluster A. (b) Mutation frequency of different genes in cluster B.

Firstly, we conducted a difference analysis of 307 ARGs between BC samples and normal samples and found that 84 genes were downregulated, and 74 genes were upregulated (Figure 1(a)). Meanwhile, 32 prognostic ARGs were screened using univariate Cox regression analysis, as shown in Table 1. We classified the aging modification patterns of 405 BC samples according to the expression of ARGs (Figure 1(b)). At the same time, we further explored the expression of ARGs to determine the optimal clustering stability (*k* = 2) and finally identified two different modification patterns (Figure 1(c)), including 211 cases in cluster A and 194 cases in cluster B. Subsequently, according to aging patterns, PCA analysis showed that BC samples could be completely distinguished (Figure 1(d)). Also, noteworthy was the results of the survival analysis, and there was more longer survival time in cluster A than in cluster B (*p* = 3.297*e* − 04), as shown in Figure 1(e). In addition, we found no significant differences in mutation analysis, which were all characterized by *TP53*, *TTN*, and *KMT2D* mutations (Figures 2(a) and 2(b)).

### 3.2. Aging-Related Patterns Regulate the Immune Microenvironment in BC Patients

**Figure 3:**
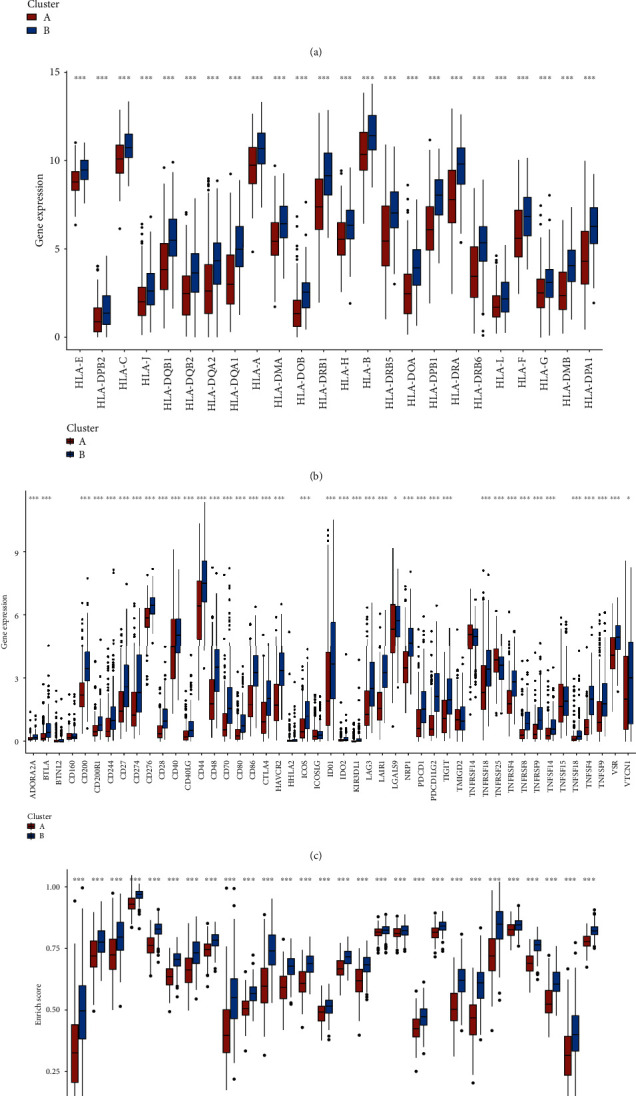
Differences in immune microenvironment based on different aging-related patterns. (a) Comparison of ESTIMATE scores, immune scores, and purity in patients with different aging-related patterns. (b) Comparison of HLA-related genes in patients with different aging-related patterns. (c) Differential expression analysis of immune cells. (d) Comparison of immune checkpoint-related genes in patients with different aging-related patterns.

We followed the algorithm in Methods to calculate the content of immune cells, stromal score, immune score, and tumor purity in whole patients with aging patterns. Compared with cluster B, patients in cluster A had higher stromal score, higher immune score, and lower tumor purity (*p* < 0.05), as shown in Figure 3(a). Excitingly, there were significant differences in immune cell content, HLA-related gene expression, and immune checkpoint-related gene expression among the different groups (*p* < 0.05), as shown in Figures 3(b)–3(d). These results may suggest that aging modifications may alter the immune microenvironment in BC tissue.

### 3.3. The Potential Biological Characteristics in Different Aging-Related Patterns

**Figure 4:**
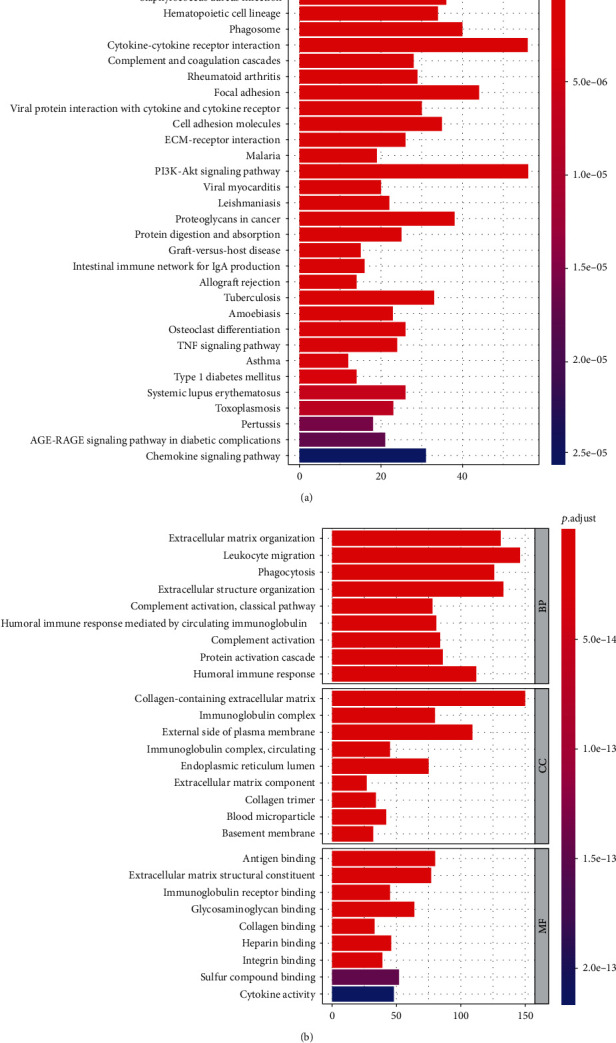
GO and KEGG enrichment analysis in different aging-related patterns. (a) KEGG enrichment analysis. (b) GO enrichment analysis, including BP, MF, and CC.

In order to explore the potential biological functions and pathways of different aging patterns, we screened out the 1221 differentially expressed gene, including 1110 upregulated genes and 111 downregulated genes. GO enrichment analysis showed that above 1221 genes were mainly related to extracellular matrix organization, etc. in the BP section, collagen-containing extracellular matrix, etc. in the CC section, and antigen binding, etc. in the MF section (Figure 4(b)). Meanwhile, GO enrichment analysis showed that above 1221 genes were mainly related to extracellular matrix organization, etc. in the BP section and collagen-containing extracellular matrix, etc. (Figure 4(a)).

### 3.4. Construction and Validation of a Risk Signature Based on Aging-Related Patterns

**Figure 5:**
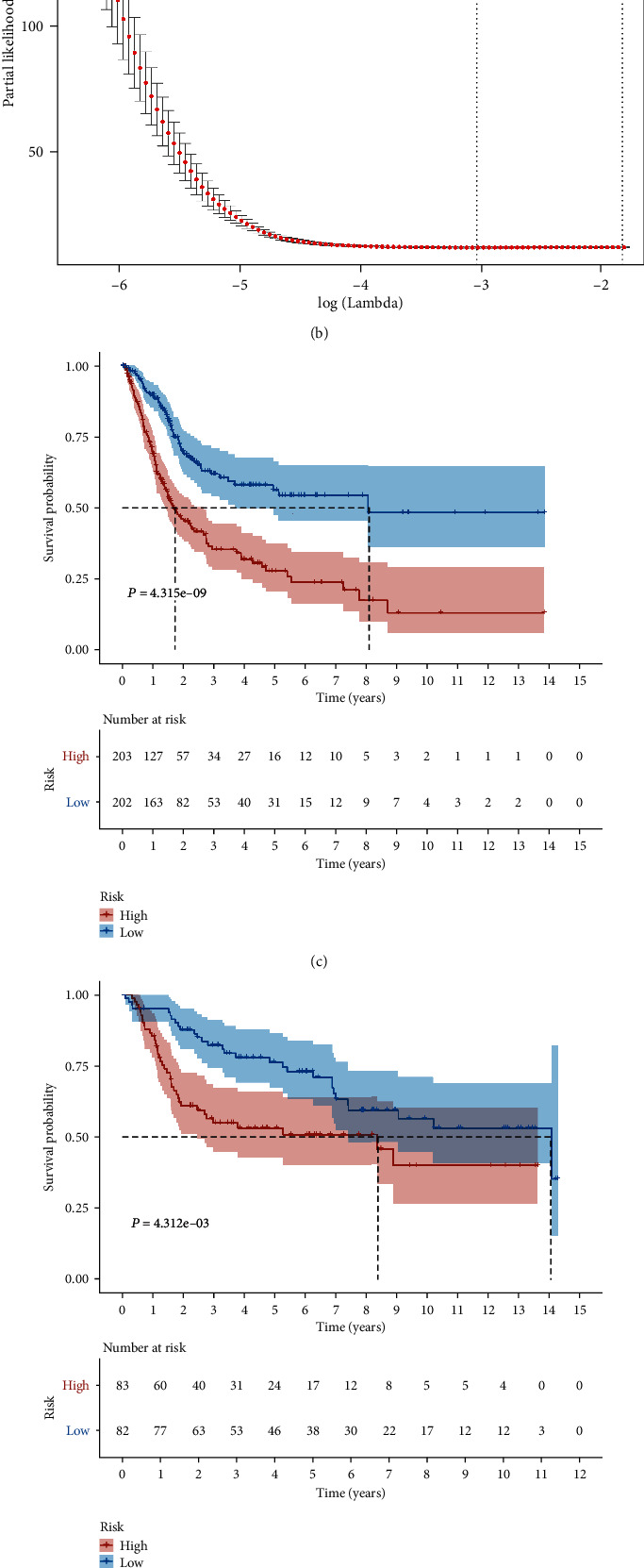
Construction and validation of a risk signature based on aging-related patterns. (a) *λ* selection plot. (b) LASSO Cox analysis of PRLs. (c) Kaplan-Meier survival analysis in TCGA cohort. (d) Kaplan-Meier survival analysis in GEO cohort. (e) ROC curve in TCGA cohort. (f) ROC curve in GEO cohort.

In order to explore the potential prognosis value in aging patterns, we screened out 944 prognosis genes in above 1221genes. To avoid collinearity in high-dimensional transcriptome data, LASSO regression was performed on these genes associated with survival (Figures 5(a) and 5(b)). Multivariate Cox regression analysis was performed for the 16 genes involved in risk signature, in which the Coef of each gene was determined. The 16 genes involved in risk signature included MXRA7, ALDH1L2, HEYL, FKBP10, TPST1, CYTL1, EPDR1, EMP1, ANXA1, FER1L4, CES1, PCOLCE2, CD3D, PTPRR, CLIC3, and CTSE. Also, noteworthy was the results of the survival analysis, and there was more shorter survival time in the high-risk group than in the low-risk gourp, and AUC values in the ROC analysis predicted 1, 3, and 5 years in TCGA cohort that are 0.713, 0.714, and 0.738, respectively (Figures 5(c) and 5(e)). To ensure the stability of this signature, we stratified the risk of primary BC patients in GSE13507 based on the above genes and divided them into risk groups according to the same cut-off value. There is no doubt that the reliability of GEO cohort for prediction in 1, 3, and 5 years is good, with AUC values of 0.606, 0.663, and 0.718, respectively (Figure 5(f)). In addition, it also had shown a robust ability to predict survival in GEO dataset (Figure 5(d)).

### 3.5. A Nomogram for Predicting Survival Status in BC Patients

**Figure 6:**
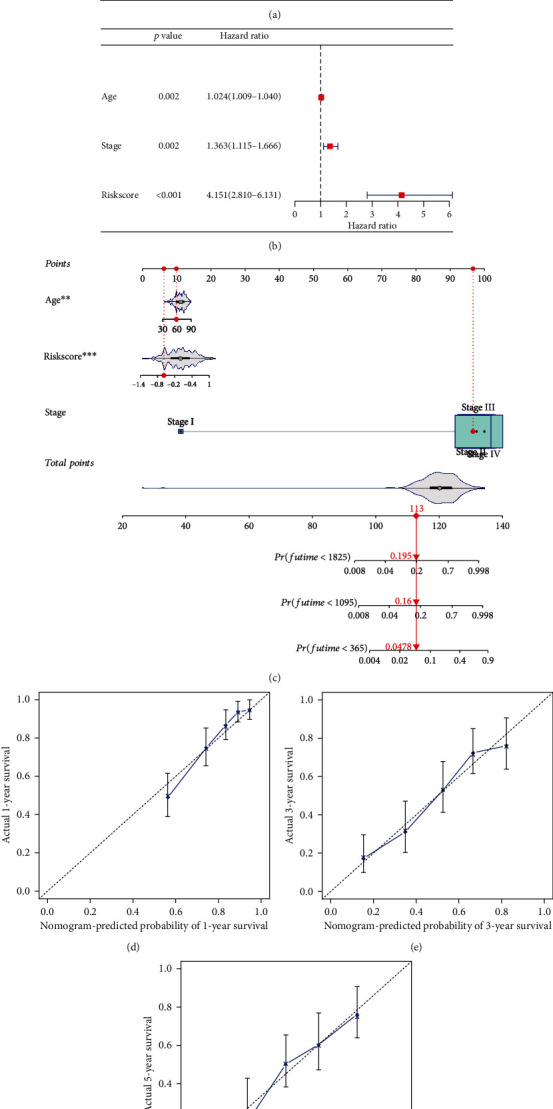
Cox regression and nomogram validation combined with clinical information. (a) A forest plot for risk score and clinicopathological factors in Cox univariate regression analysis. (b) A forest plot for risk score and clinicopathological factors in Cox multivariate regression analysis. (c) A nomogram based on risk score, age, and stage. (d) Calibration curves of 1, 3, and 5 years.

To further investigate the independent prognostic value of this prognostic signature, Cox regression analysis showed that risk score was an independent prognostic factor, as was age and stage (Figures 6(a) and 6(b)). We combined indicators in Cox regression analysis to construct the visual prognostic model-nomogram, as shown in Figure 6(c). Moreover, the calibration curve of the nomogram showed that the prediction curves are close to the standard curve in TCGA cohort, which indicates that the predicted survival rate is closely related to the actual rates at 1, 3, and 5 years, as shown in Figures 6(d)–6(f).

### 3.6. Differences in Immune Microenvironment Based on Different Risk Subgroups

**Figure 7:**
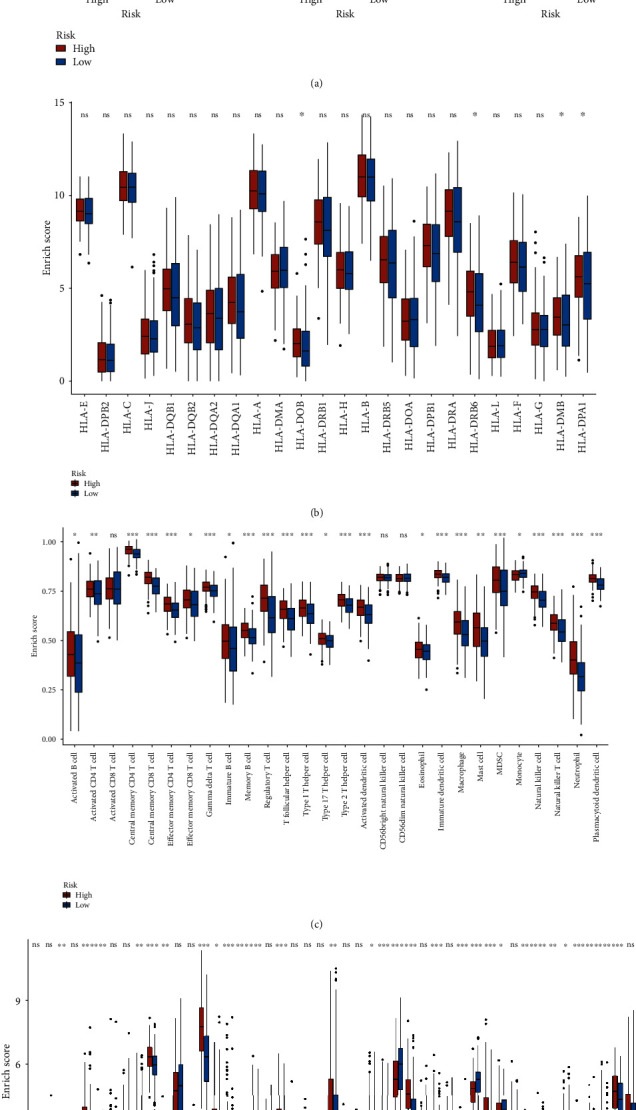
Differences in immune microenvironment based on different risk subgroups. (a) Comparison of ESTIMATE scores, immune scores, and purity in patients with different risk subgroups. (b) Comparison of HLA-related genes in patients with different risk subgroups. (c) Differential expression analysis of immune cells. (d) Comparison of immune checkpoint-related genes in patients with different risk subgroups.

We followed the algorithm in Methods to calculate the content of immune cells, stromal score, immune score, and tumor purity in all patients with different risk subgroups. Compared with the low-risk group, patients in the high-risk group had higher stromal score, higher immune score, and lower tumor purity (*p* < 0.05) (Figure 7(a)). However, unlike the aging pattern, differences in the expression of only a few HLA-related genes, such as HLA-DOB, HLD-DRB6,HLA-DMB, and HLA-DPA1, were found between risk groups (*p* < 0.05) (Figure 7(b)). Excitingly, there also were significant differences in immune cell content and immune checkpoint-related gene expression among the different groups (*p* < 0.05), as shown in Figures 7(c) and 7(d). These results may suggest that risk subgroups based on aging pattern may distinguish the immune microenvironment of BC tissues.

### 3.7. Construction of PPI Network and Exploration of a Hub Gene

**Figure 8:**
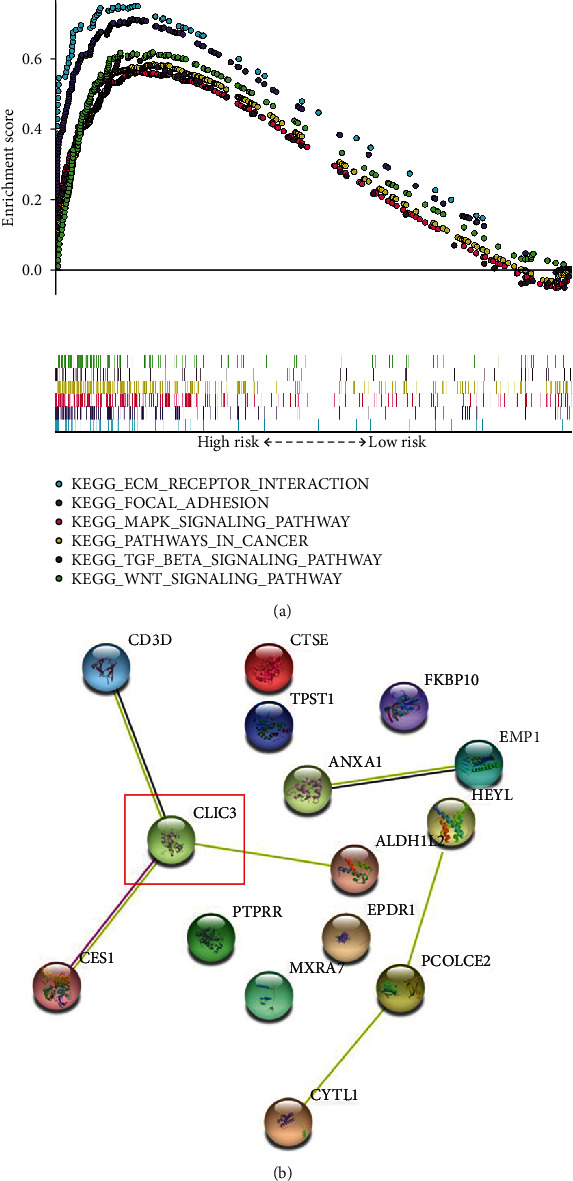
GSEA analysis and PPI network in risk groups. (a) GSEA analysis in the high-risk gourp. (b) A PPI network in 16 genes participating in risk signature.

In order to study the biological pathways that may differ in different risk groups, we used GSEA enrichment analysis to find that the high-risk group may be associated with ecm receptor interaction, focal adhesion, mapk signaling pathway, wnt signaling pathway, etc. (Figure 8(a)). In addition, we performed genes involved in risk signature that were imported into a STRING database (confidence = 0.900). Finally, we then selected the *CLIC3* with the highest number of sides as the hub gene (Figure 8(b)).

### 3.8. Vitro Validation

**Figure 9:**
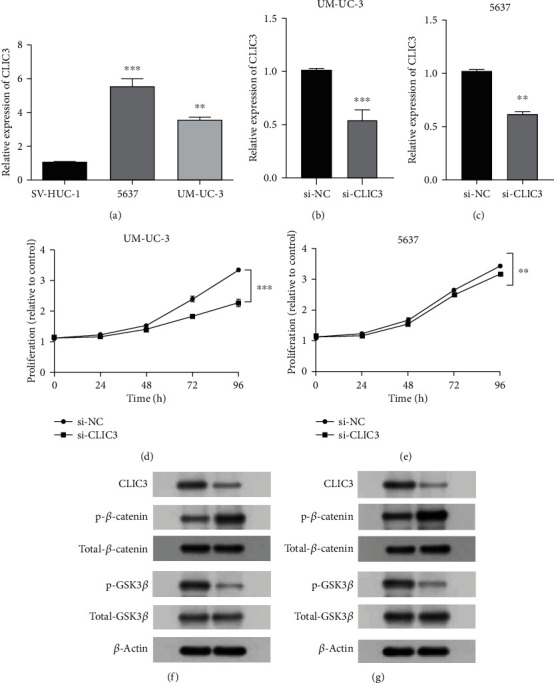
Vitro validation. (a) Relative expression of *CLIC3* mRNA in normal and tumor cell lines. (b) Relative expression of *CLIC3* mRNA in UM-UC-3 cell lines transfected with si-*CLIC3*. (c) Relative expression of *CLIC3* mRNA in 5637 cell lines transfected with si-*CLIC3*. (d) CCK8 assays in UM-UC-3 cell lines transfected with si-*CLIC3.* (e) CCK8 assays in 5637 cell lines transfected with si-*CLIC3.* (f) Western blot analysis of CLIC3, p-*β*-catenin, total-*β*-catenin, p-GSK3*β*, and total-GSK3*β* expression in UM-UC-3 cell line after transfection. (g) Western blot analysis of CLIC3, p-*β*-catenin, total-*β*-catenin, p-GSK3*β*, and total-GSK3*β* expression in 5637 cell line after transfection. ^∗^*p* < 0.05, ^∗∗^*p* < 0.01, ^∗∗∗^*p* < 0.001.

To further validate the hub gene of the PPI network, we detected expression level of CLIC3 mRNA in BC cell lines. The results showed that the expression of CLIC3 was upregulated in tumor cell lines compared to SV-HUC-1, as shown in Figure 9(a). In addition, si-CLIC3 and si-NC were transfected in 5637 and UM-UC-3 cells, respectively, and qRT-PCR and Western blot were used to detect the protein expression of AGTRAP. It was found that the CLIC3 expression was downregulated in BC cell lines with transfection, as shown in Figures 9(b) and 9(c). Similarly, CCK-8 assays showed that BC cell proliferation was inhibited after transfection with CLIC3, as shown in Figures 9(d) and 9(e). In addition, the result of GSEA enrichment analysis showed that Wnt signaling pathway is activated in the high-risk group; so, we performed Western blot assays in BC cell lines transfected with *CLIC3* siRNA to detect this pathway. Western blot analysis revealed that p-*β*-catenin levels were increased, and p-GSK3*β* levels were decreased in 5637 and UM-UC-3 cells after CLIC3 silencing. The ratio of p-*β*-catenin to total *β*-catenin was increased, while the ratio of p-GSK3*β* to GSK3*β* was decreased, suggesting the shutdown of Wnt/*β*-catenin signaling, as shown in Figures 9(f) and 9(g).

## 4. Discussion

In biologically speaking, ageing is a natural process that cannot be avoided over time. It manifests itself such as degenerative changes in structure and a decline in function [5]. Aging has been identified as an independent risk factor for the majority of common cancers, including BC [16]. Furthermore, aging may have a tumor-suppressing effect, as tumors that are in a state of senescence-induced growth arrest slow down their growth [5]. Because of the importance of aging-related genes, ageing indicators may also have the ability to predict prognisis in cancer patients.

We performed a PPI network analysis on 17 aging-associated genes included in the risk signature, and *CLIC3* was experimentally validated. To our knowledge, *CLIC3* belongs to the intracellular chloride channel family [17], and it is overexpressed in a variety of tumors, resulting in a poor prognosis for patients [18]. CLIC3 has been demonstrated to have a role in invasion and metastasis in breast cancer cell line, and the overexpression of CLIC3 in oestrogen receptor-negative predicts a poor prognosis [19]. Furthermore, CLIC3 and Rab25 work together to promote cancer progression [20]. Our findings imply that more research into the involvement of *CLIC3* in cellular senescence is warranted. Although there is currently a bioinformation-based study on the role of CLIC3 in bladder cancer, the study only conducted a simple expression difference analysis and clinical correlation of CLIC3 [15]. In the validation part of our study, we further explored the protein expression of CLIC3 and the change of Wnt pathway in BC cell lines after CLIC3 silencing.

To construct aging-related patterns and investigate their impact on the immunological microenvironment, pharmaceutical sensitivity, and survival status in BC patients, we utilized differential ARGs in the consensus clustering method. Following that, 17 ARGs were chosen from the differential genes in aging-related patterns to construct a risk signature for TCGA cohort. There are still some limitations of our study that are worth noting. The bioinformatic results, for starters, have been validated using TCGA and GEO samples. However, we were unable to conduct a second external validation, because we lacked the sufficient funding to sequence BC patients in our hospital. Second, we only used PPI network to corroborate our findings for the hub gene, and we will need to conduct more experiments in the future to confirm our conclusion.

In conclusion, we have developed a risk signature based on ARGs and validated the hub gene. Therefore, the findings based on this study are useful for promoting individualized immunotherapy and survival prediction in BC patients.

## 5. Conclusion

We provided robust evidences that aging patterns based on ARGs can guide targeted therapy and survival prediction in BC patients.

## Figures and Tables

**Table 1:** Univariate Cox regression analysis of differential expression ARGs in patients with bladder cancer.

ARGs	HR	HR.95L	HR.95H	*p* value
AGTR1	1.1551	1.0081	1.3234	0.0379
EGR1	1.1502	1.0452	1.2658	0.0042
GHR	1.2555	1.0760	1.4648	0.0038
A2M	1.1255	1.0166	1.2461	0.0227
FGFR1	1.1679	1.0635	1.2824	0.0012
CTGF	1.1304	1.0398	1.2288	0.0040
NUDT1	1.2603	1.0175	1.5611	0.0341
JUN	1.1888	1.0463	1.3507	0.0080
PDGFRA	1.2099	1.0778	1.3583	0.0012
PYCR1	1.1704	1.0482	1.3068	0.0051
ELN	1.1368	1.0501	1.2306	0.0015
SIRT6	0.6340	0.4577	0.8782	0.0061
RAE1	1.3194	1.0005	1.7399	0.0496
NGF	1.2540	1.1046	1.4235	0.0005
IGF1	1.3595	1.1103	1.6646	0.0030
EFEMP1	1.1450	1.0727	1.2221	0.0000
MYC	1.1543	1.0445	1.2756	0.0049
NGFR	1.0914	1.0030	1.1877	0.0425
LMNA	1.2945	1.0409	1.6098	0.0203
TFDP1	1.2020	1.0031	1.4404	0.0462
PLAU	1.1024	1.0084	1.2052	0.0320
POLB	0.7633	0.6338	0.9193	0.0044
FOXO3	1.2321	1.0034	1.5130	0.0463
APEX1	1.4098	1.0529	1.8877	0.0211
NCOR1	1.2654	1.0134	1.5802	0.0378
STAT5A	0.8325	0.7013	0.9882	0.0361
PAPPA	1.3585	1.1414	1.6170	0.0006
GRN	1.2490	1.0109	1.5432	0.0394
PDGFRB	1.2099	1.0871	1.3465	0.0005
PRDX1	1.5245	1.2226	1.9008	0.0002
EIF5A2	1.2015	1.0373	1.3918	0.0144
ERCC5	0.6840	0.5056	0.9253	0.0138

## Data Availability

The following information was supplied regarding data availability: data is available at the TCGA (https://portal.gdc.cancer.gov/) and GEO database (https://www.ncbi.nlm.nih.gov/geo/).
